# Current and Potential Tree Locations in Tree Line Ecotone of Changbai Mountains, Northeast China: The Controlling Effects of Topography

**DOI:** 10.1371/journal.pone.0106114

**Published:** 2014-08-29

**Authors:** Shengwei Zong, Zhengfang Wu, Jiawei Xu, Ming Li, Xiaofeng Gao, Hongshi He, Haibo Du, Lei Wang

**Affiliations:** 1 School of geographical sciences, Northeast Normal University, Changchun, China; 2 College of Urban and Environment Sciences, Shanxi Normal University, Shanxi, China; 3 School of geography, Beijing Normal University, Beijing, China; 4 School of Natural Resources, University of Missouri-Colombia, Columbia, Missouri, United States of America; Chinese Academy of Sciences, China

## Abstract

Tree line ecotone in the Changbai Mountains has undergone large changes in the past decades. Tree locations show variations on the four sides of the mountains, especially on the northern and western sides, which has not been fully explained. Previous studies attributed such variations to the variations in temperature. However, in this study, we hypothesized that topographic controls were responsible for causing the variations in the tree locations in tree line ecotone of the Changbai Mountains. To test the hypothesis, we used IKONOS images and WorldView-1 image to identify the tree locations and developed a logistic regression model using topographical variables to identify the dominant controls of the tree locations. The results showed that aspect, wetness, and slope were dominant controls for tree locations on western side of the mountains, whereas altitude, SPI, and aspect were the dominant factors on northern side. The upmost altitude a tree can currently reach was 2140 m asl on the northern side and 2060 m asl on western side. The model predicted results showed that habitats above the current tree line on the both sides were available for trees. Tree recruitments under the current tree line may take advantage of the available habitats at higher elevations based on the current tree location. Our research confirmed the controlling effects of topography on the tree locations in the tree line ecotone of Changbai Mountains and suggested that it was essential to assess the tree response to topography in the research of tree line ecotone.

## Introduction

Tree line is considered as an important indicator of terrestrial ecosystems to climate change because it is sensitive to environmental change. It has been proved that tree line species have advanced in the past decades, regardless they are in arctic or in the alpine regions [1], [2]. Numerous studies show that the temperature is responsible for causing the upward shift of trees as average temperatures have raised globally over the past century [3], [4], [5]. Increasing temperature may promote the growth of plant species at the tree line. However, the responses of tree species around tree line to the climate warming differ in different regions. Some researches show that tree line species are particularly responsive to changes in summer temperature [6], whereas others suggest that winter temperature may have important effects [7], [8]. Variations in average and seasonal temperature changes may influence the establishment of tree recruitments [9]. However, air temperature may not be the only dominant factor controlling tree growth at tree lines. According to Liang et al. (2011), Smith fir (*Abies georgei* var. *smithii*) does not show a significant upward movement, despite warming of the Tibetan Plateau. The warming in the past 200 year is already having a significant impact on the population density of the trees, but not on the position of the Smith fir tree line [10]. Furthermore, soil temperature has greater influences than air temperature on tree growth at tree line position [11]. The effects of temperature on the tree locations around tree line cannot be ignored. However, in mountainous regions, topography may control local environments including microclimates, soil properties, and disturbances [12] and exerts a strong influence on vegetation distribution and succession in tree line ecotone [13], [14]. On one hand, topographic shelters may be important controls in determining where pioneer tree species can establish and persist in the initial stages from upslope tree migration to alpine tundra [15]. On the other hand, the effect of varying topography on site conditions combined with natural disturbances may override the effects of temperature [16]. Therefore, it is essential to assess the response of tree species to topography in tree line ecotone.

Tree line ecotone in Changbai Mountains has undergone large changes over the past decades, in particular, on the northern and western sides of the mountains. Previous researches have mainly focused on tree line changes on the northern side. Most of these studies investigated the effects of climate warming on trees and attributed the main effects to the temperature change [17]. According to Wang and Liu (2011), trees cannot reach its potential altitude owing to the asynchronous changes in heat and water at high elevations, which also confirms the effects of climate change [18]. Physiological drought in *Betula. ermanii* during its growing season, caused by variation in water and heat along the altitudinal gradient, restricted the distribution of the *B. ermanii* to higher elevations in the Changbai Mountains [19]. Apart from climate change, some experts have also expressed concerns about the effects of other factors including the shape of forest boundaries (concave and convex boundary) and soil properties [20], [21]. However, limited researches have been conducted on the topography of the tree line ecotone in Changbai Mountains. It was notable that the important climatic factors for forests at the tree line ecotone are temperature and solar radiation, which are exactly influenced by the topography [1]. Therefore, the examination of tree locations in tree line ecotone by topographic variables could help explain the variations in tree line ecotone of Changbai Mountains.

Vegetation on the western side of the mountains has also experienced large changes during the past decades. A large windthrow occurred in the tree line ecotone in 1986, which almost destroyed the *B. ermanii* forests [22]. In addition, Herbaceous species from *B. ermanii* forest, represented by *Deyeuxia angustifolia*, have invaded the alpine tundra and led to severe impacts on the alpine tundra ecosystem [23]. Invasion of *D. angustifolia* represented the invasion of the Mountain birch forest [23]. However, it is still not clear whether trees on the western side would move up to high elevations. Therefore, there is an urgent need to study the current state of tree locations on the western side to understand its future changes. The results could help to facilitate the interpretation of changes in alpine tree line ecotone.

In this study, we hypothesize: 1) topographic differences are responsible for the differences in tree locations on northern and western sides of the mountains because the two sides have distinct topographic characteristics; and 2) habitats above the current tree line are available for tree species to establish. Our specific objectives are to 1) interpret the current tree locations; 2) evaluate the controlling effects of topography on the tree locations; and 3) predict the potential habitat of trees locations in tree line ecotone on the northern and western side of the Changbai Mountains.

### Study area

**Figure 1:**
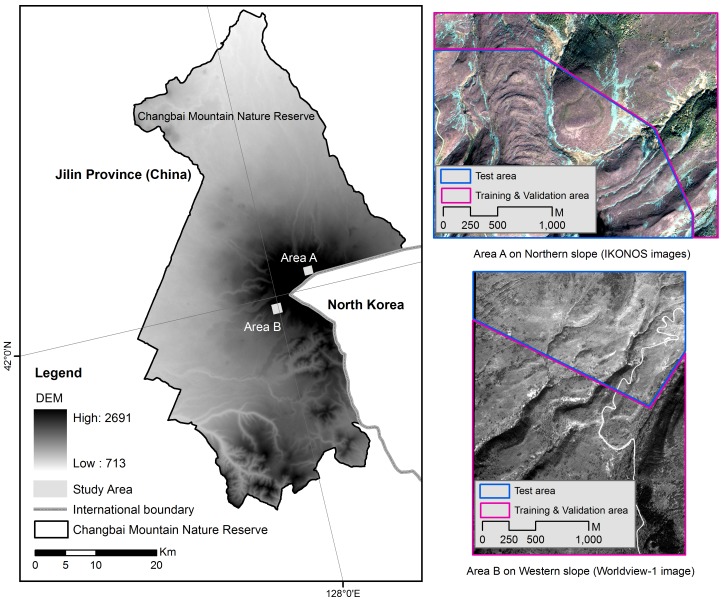
Location of the study areas in Changbai Mountains Nature Reserve in Jilin province, Northeast China. DEM (Digital elevation model) value ranges from 713 to 2681. Area A shown by the IKONOS image (False color image created by combining the blue band, green band, and red band) is on the northern slope. Area B shown by the WorldView-1 image is on the western slope. Red area represented the training and validation area. Blue area represented the test area which is used for the prediction of tree locations.

**Figure 2:**
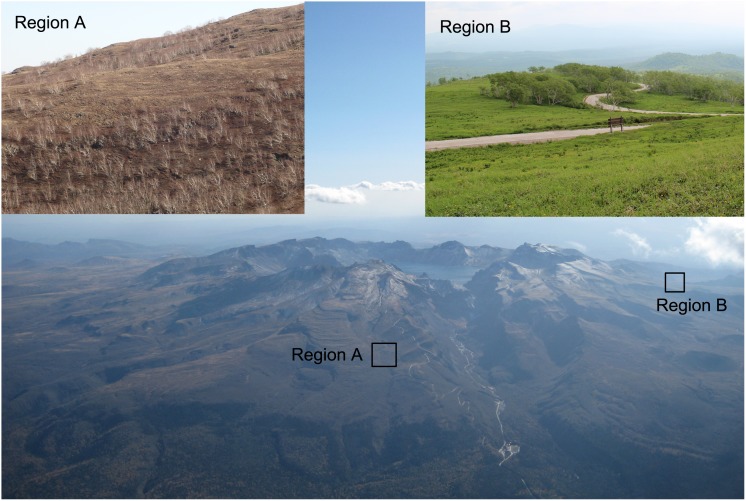
Aerial photograph of the volcanic cone in the Changbai Mountains. The photo was taken from the northern side on October 2010. Region A is on the northern slope. It can be seen that trees in the northern side gradually move upward to the high elevations. Region B is on the western side. It can be seen that tree line position in western side is relatively stable.

The study area (41°58′–42°02′N, 127°58′–128°05′E) is located in the Changbai Mountains National Nature Reserve (CMNNR) in Jilin province, Northeast China (Fig. 1). The CMNNR has vertical forest zones along the altitudinal gradient, in particular, on the northern side of the mountains. The tree line surrounds the volcanic cone at different altitudes on the four sides. The main tree species in tree line ecotone include *B. ermanii*, *Larix olgensis*, and *Alnus tinctoria*. On the northern side, *B. ermanii* has invaded into the alpine tundra zone, which led to the expansion of the tree line over the last few decades [17]. In contrast, there has been no expansion of the trees on western side (Fig. 2). The tree line position on the western side has been steady over the past decades. The study area is not affected by anthropogenic activity. Therefore, it is an ideal area for studying the tree locations, in particular, in the temperate zone of Northeast China. We defined the tree line ecotone as an area of transition spanning from close canopy forests to open area where trees sparsely distributed. We used tree line ecotone to simply refer the area where we made predictions of current and potential tree locations. The definition of tree line ecotone itself did not influence in any shape or form on our results.

The climate of the study area is characterized low temperature, heavy precipitation and a short growing season. Annual mean temperatures in the growing season (June to September) range from 3.37 to 8.82°C (mean temperature is 5.87°C). Annual average precipitation ranges from 700 to 1,400 mm [24]. The mean annual radiation is 506.6 J.cm^−2^.a^−1^. The mean annual sunlight duration is 2295 h. Harsh environment is harmful to plant organism that limits the photosynthesis capability and vegetative growth [25]. Therefore, the growth rate of alpine trees is very slow. The thin soil (only about 10–25 cm depth) in study area is the alpine tundra meadow soil.

The two areas chosen for this study (Fig. 1) are typical and representative areas on the two sides of the mountains, which is conducive for the comparative analysis of the differences in tree locations between the northern and western side. The area on the northern side covers approximately 5.36 km^2^ (Fig. 1). The western area covers approximately 5.33 km^2^. The red area in Figure 1 represents the training and validation area with 20% of this area used specifically for training and the left used as the validation area. Detailed information can be found in the description of the sampling in the Materials and Methods section. The blue area represents the test area, which is used for the prediction of the potential habitat of trees.

## Materials and Methods

The field work assisted by the Scientific Research Academy of the Changbai Mountains (http://cbs.jl.gov.cn/ResearchWeb/main.aspx) was permitted by Administration Commission of Changbai Mountains Nature Reserve (http://www.cbs.jl.gov.cn/weball/main.aspx#). The field studies conducted on the west side (127°58′56.827″E–128°0′22.129″E, 41°58′10.543″E–41°59′36.287″) and north side (128°4′2.67″E–128°5′54.832″E, 42°1′15.245″E–42°2′21.036″E) of the mountains did not involve endangered or protected species.

### Data

Studies of individual tree location require high spatial resolution satellite images. Tree lines in Changbai Mountains have a diffused form, which indicates that many tree locations would not be detected if low resolution images were used. Therefore we use the high-resolution images to get the accurate tree location data.

The satellite image data used in this study include IKONOS data for the northern side and WorldView-1 for the western side. The IKONOS data consist of one panchromatic image at 1-m resolution and four multispectral images at 4-m resolution, which are acquired on 2002-09-20. The panchromatic image covers a spectral range from the visible red to the near-infrared region (450–900 nm). The multispectral images covers four bands including the visible blue (445–516 nm), green (506–595 nm), red (632–698 nm), and near-infrared (770–888 nm) bands, respectively. The WorldView-1 data consists of one panchromatic image at 0.5-m resolution with a spectral range of 400–900 nm, which is acquired on 2009-10-09. We consider that the differences in acquired time of the two image data would not affect the comparative results of the two sides because there was little change of tree locations during the period of 2002–2009. The two image data are preprocessed using standardized procedures of radiometrically correction, sensor correction, and geometrically correction.

**Figure 3:**
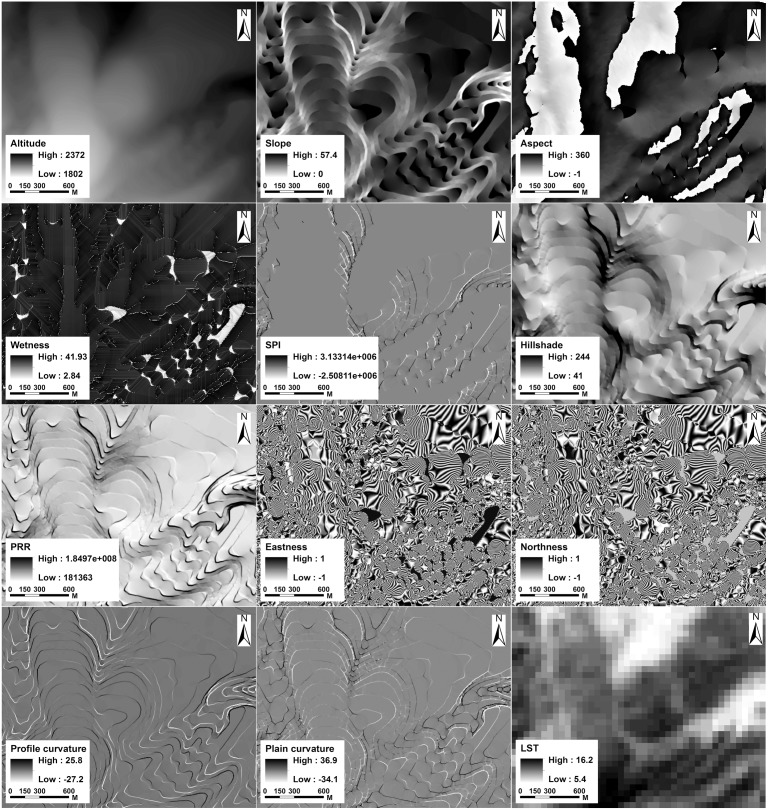
Topographic variables of the northern side derived from the digital elevation model. PRR represents potential relative radiation which sums daily values over the growing season. SPI represents snow potential index which indicates the snow accumulation in topography. LST represents the land surface temperature.

**Figure 4:**
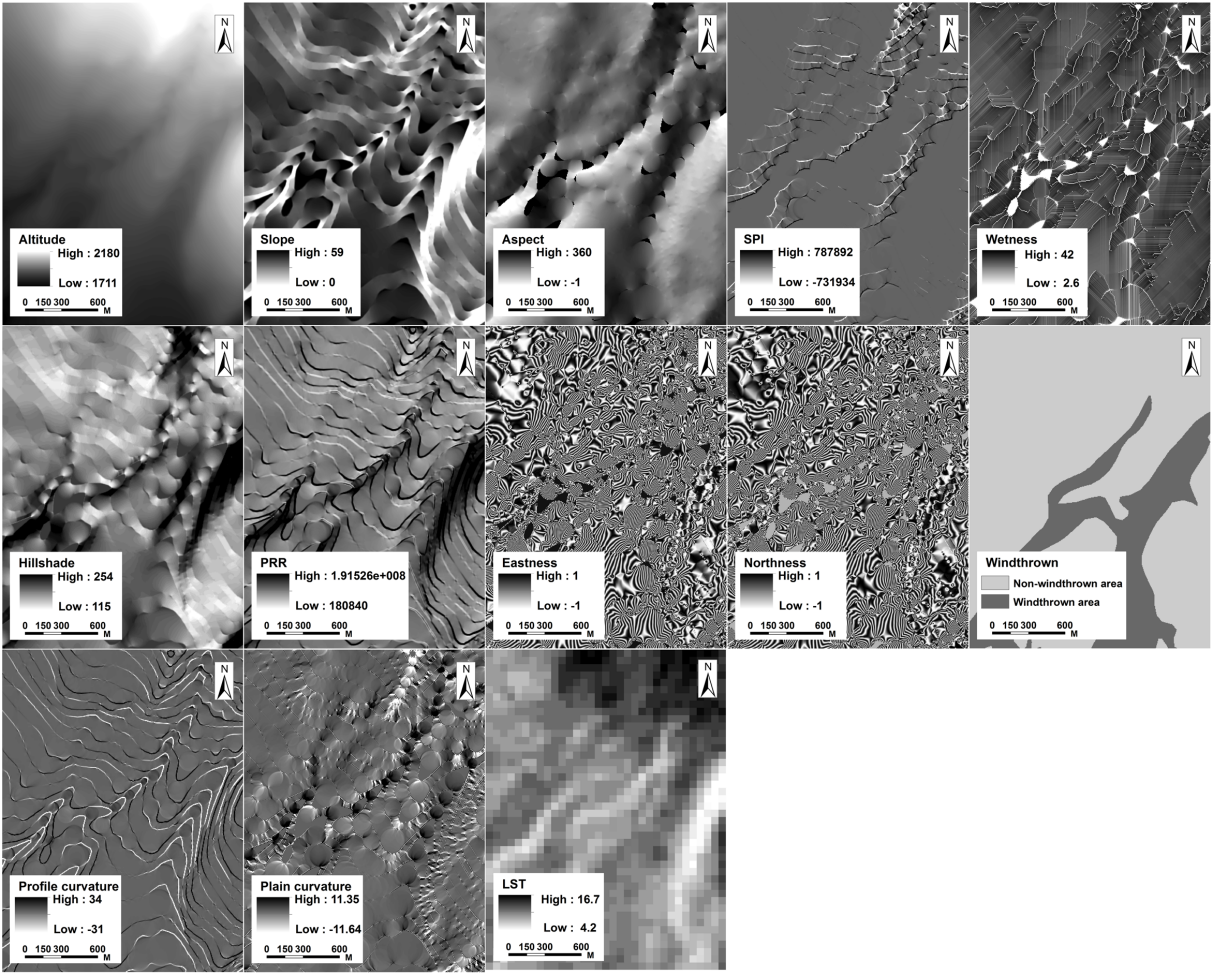
Topographic variables of the western side derived from the digital elevation model. PRR represents potential relative radiation which sums daily values over the growing season. SPI represents snow potential index which indicates the snow accumulation in topography. LST represents the land surface temperature.

The DEM (Digital elevation model) data are derived from the 1: 50 000 topographic maps (Data source: National Administration of Surveying, Mapping and Geoinformation (http://chj.jl.gov.cn/article/hdjl/lyzx/?siteid=jilin). The cellsize is confined to 5 m. The DEM error is defined as the elevation difference between the constructed DEM and the corresponding true surface [26]. To eliminate the DEM error, we collected 205 GPS positions as ground truth points. We used the Real-Time Kinematic (RTK) GPS equipment, which can deliver almost instantaneous point coordinates with centimetre-scale accuracy [27]. A reference station of RTK GPS was set up at the Tianchi weather station (128°04′02″E, 42°01′40″N) in the Changbai Mountains with known coordinates. Then a receiver was used to record the ground truth points. After the field survey, the RTK GPS data were imported into ArcGIS for DEM quality assessment and correction. Based on this correction, the height error was <0.25 m. However, the DEM still has some negative values and contained terraces. We therefore used ArcGIS functions to remove these values and fill the sinks [28]. Finally, a high quality DEM was prepared to derive the topographic variables (Table 1). Approximately 14 topographic variables (14 for the northern side and 15 for the western side) were selected based on our hypothesis that the tree locations may be determined by a variety of topographic factors. These topographic indices, such as the Wetness Index, have significant ecological implications for explaining certain ecological processes. The topographic variables (Figs. 3 and 4) used in this study have been previously applied in several studies [12], [29], [30]. All the indices were derived from the DEM data. The wetness index is based on the assumption that topography controls the movement of water in terrains with slopes, and thus, it regulates the spatial pattern of soil moisture. High values of the wetness index are found in converging, flat terrain. Low values are typically found in steep, diverging areas. PRR (Potential relative radiation) sums hourly estimates of clear-sky radiation over a day and then sums daily values over the growing season. Each point estimate accounts for topographic shading by surrounding landscape features. Hillshade index reflects the sunlight conditions in the mountainous area, which is created from DEM raster by considering the illumination angle and shadows. High value represents the shadow areas whereas low value means the bright area. The SPI (snow potential index) represents the potential for snow accumulation at specific sites. Higher values of the index were obtained on slopes that were leeward (i.e. closer to northeast exposure), concave, and at higher elevations. In addition, it was evident that vegetation on the western side had experienced a severe typhoon disturbance in 1986, which resulted in wide-spread windthrown areas. Therefore, a raster of the windthrown areas was produced as the natural disturbance index (Fig. 4) for the western side. Land Surface Temperature (LST) data which represents the temperature condition were derived from Landsat ETM+ data using the method from Li et al (2004) [31]. Corresponding to the acquire time of the IKONOS (September 20, 2002) and Worldview-1 (October 9, 2009) data, we collected two scenes of cloud and snow free Landsat ETM+ images on August 25, 2002 and September 29, 2009. We assumed that there was little change of temperature distribution pattern along altitudinal gradient on the alpine tundra at the end of growing season (late August to early October). Therefore, the LST data could be used as a variable concerning temperature in the process of constructing the model. All the Landsat ETM+ data were preprocessed using standardized procedures of radiometrically correction, sensor correction, and geometrically correction.

**Table 1:** Topographical indices used in this study.

Variable	Description	Calculation	Ecological meaning
Altitude	Altitude above sea level (m)		Temperature, moisture
Slope	Slope angle (degrees)		Solar radiation, soil erosion, moisture
Aspect	Aspect angle (0° to 360°)		Temperature, wind
Eastness	Aspect east-west (1 to –1)	sin(aspect)	Morning/afternoon solar radiation
Northness	Aspect north-south (1 to –1)	cos(aspect)	Summer/winter solar radiation
Plan curvature	Curvature perpendicular to slope	–1 = concave, 1 = convex	Solar radiation, wind, moisture
Profile curvature	Curvature in slope direction	–1 = convex, 1 = concave	Moisture
Hillshade	Hillshade surface	hillshade with sunelevation angle of 45°and sun azimuth angle of 315°	Solar radiation
PRR	Potential relative radiation	sums daily values over thegrowing season [31]	Solar radiation potential
SPI	Snow potential index	Snow accumulationin topography [12]	Temperature, moisture
Surface roughness	Roughness of regional area	1/cos(slope×π/180) [46]	wind, moisture
Surface undulation	Undulation of regional area	Max(regional altitude)−Min(regional altitude) [46]	wind, moisture
Wetness	Topographic wetness index	ln(A_s_/tan β) [32]	moisture
Windthrow index	Windthrown area by typhoon	0 = non-windthrow,1 = windthrow [45]	Typhoon disturbance
LST	Land surface temperature	Derived from LandsatTM/ETM+ images [33]	Temperature

### Data preparation

For IKONOS images, a high quality one-meter resolution pan-sharpened image was first produced by Gram-Schmidt spectral sharpening. Then, a false color composite image was created by combining the blue band, green band, and red band. Afterwards, tree locations were identified manually with interactive interpretation procedures, supplemented by different image transformations, such as vegetation indices which can minimize shadow and atmospheric condition effects [32]. The tree locations of the WorldView-1 image were identified using unsupervised classification with the ISODATA (Iterative Self-Organizing Data Analysis Technique) method. The results were manually modified with ground truth data. The ground truth data were divided into two parts. One section was used to generate the ROI (region of interest) data. The other was used to examine the accuracy of the classification result. The tree species examined in the study area included *B. ermanii* and *L. olgensis*. On the northern side, there were few *A. tinctoria* distributed in the tree line ecotone. The final classification map was a binary (0 = tree absence; 1 = tree presence). GCPs (Ground control points) were important for accuracy assessment of classification map, which were also obtained using GPS during August 2012. We obtained 183 GCPs of tree sites on the northern side, and 273 GCPs on the western side. Collection of GCPs for assessing the accuracy of the image classification and DEM was conducted simultaneously. The classification map of the IKONOS image and the WorldView-1 image were resampled at a resolution of 5 m using bilinear interpolation to match the resolution of the DEM and the derived variables. All image data were converted from disparate sources to a common format defined in Arc/Info grid with Gauss-Kruger projection and Krasovsky 1940 spheroid.

### Sampling method

Species distributional data often display spatial autocorrelation [33]. Consequently, in the modeling process, the spatial autocorrelation may bias parameter estimates. To avoid the autocorrelation effect, a useful solution is to increase the sample distance and decrease the correlation between points [12]. We used the “Sample” tool of the ArcGIS toolbox to obtain the sample data used for model construction. Sample distance ranged from 1 to 20 pixels. Then, the sample data was imported into SPSS (SPSS Inc., Chicago, IL, USA) to check the spatial autocorrelation using the method of “Time Series Analysis”. We found that the autocorrelation coefficient was below 0.530 after the sample distance was increased to 4 pixels apart. Therefore, the optimal sample distance was determined as 4 pixels.

### Logistic regression model

In this study, logistic regression model was applied to predict the tree/non-tree locations based on the topographic variables:
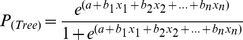
(1)


In the equation, *a* was the constant; *b_1-n_* were the coefficients of the variables (*x*_1-n_). In this study, the included variables were shown in Table 1. *P* was a probability value from 0 to 1.0, which was the probability value of the tree locations.

Before importing the topographic variables into the logistic regression procedure, the multicollinearity of all the explanatory variables which would bias the model estimate was checked. The linear correlations of the variables were tested among the variables. The correlation matrix (Table 2) among the variables showed that, on the western side, the surface undulation was highly correlated with the surface roughness (0.8774) and slope (0.9240), and that the surface roughness was highly correlated with slope (0.9179). On the northern side, the surface undulation was highly correlated with the surface roughness (0.8627) and slope (0.8889), and the surface roughness was highly correlated with slope (0.8483). Therefore, surface roughness and surface undulation were excluded. For the regression, ‘binary logistic regression’ was used with the forward stepwise algorithm based on a maximum-likelihood ratio test. We ran the model with all the cut-off points between 0 and 1.0 with intervals of 0.1, and obtained correct classification rates for presences, absences and both. The cut-off value of *P* was finally set to 0.04 for the western side and 0.06 for the northern side. Values above this threshold were considered as tree presences, while values below the threshold were considered as tree absences.

**Table 2:** Correlation matrix of Topographic indices of western northern side.

	western side																		
	Surfaceundulation	Profilecurvature		Plancurvature		Windthrowindex		Northness		Wetness	Eastness	Altitude	SPI	PRR	Surfaceroughness	Slope	Aspect	Hillshade	LST
Surface undulation	1	0.0043		0.0332		0.0668		0.0306		0.3104	0.0598	0.3468	0.0247	0.3707	**0.8774**	**0.9240**	0.3511	0.3656	–0.1607
Profile curvature		1		0.3876		0.0492		0.0126		0.0165	0.0058	0.0346	0.5442	0.2021	0.0007	0.0027	0.0062	0.0163	–0.0658
Plan curvature				1		0.1275		0.0059		0.0694	0.0033	0.1167	0.3930	0.0045	0.0407	0.0254	0.0086	0.0242	–0.0048
Windthrow index						1		0.0017		0.1101	0.0084	0.2936	0.0396	0.1875	0.0712	0.0296	0.0679	0.2557	–0.1115
Northness								1		0.1116	0.0228	0.0147	0.0070	0.0122	0.0315	0.0472	0.0726	0.0082	0.1414
Wetness										1	0.1706	0.1695	0.0247	0.0227	0.2197	0.3912	0.5477	0.0881	0.2397
Eastness											1	0.0187	0.0091	0.0037	0.0483	0.0787	0.1300	0.0260	0.2224
Altitude												1	0.0286	0.0749	0.2339	0.3151	0.0856	0.2057	0.4870
SPI													1	0.1079	0.0112	0.0106	0.0046	0.0073	0.2516
PRR														1	0.4302	0.3197	0.3249	0.5123	0.1714
Surface roughness															1	**0.9179**	0.3246	0.5136	–0.1299
Slope																1	0.3766	0.3050	–0.1020
Aspect																	1	0.0317	–0.4717
Hillshade																		1	0.1737
LST																			1
	**northern side**																		
	**Surface** **undulation**		**Aspect**		**Slope**		**Surface** **roughness**		**PRR**		**SPI**	**Eastness**	**Hillshade**	**Northness**	**Wetness**	**Altitude**	**Plan** **curvature**	**Profile** **curvature**	**LST**
Surface undulation	1		0.2757		**0.8889**		**0.8627**		0.3019		0.0100	0.0276	0.3470	0.0210	0.2995	0.2660	0.0015	0.0080	–0.0131
Aspect			1		0.3086		0.2419		0.2015		0.0009	0.0310	0.0264	0.0095	0.1850	0.0332	0.0026	0.0045	–0.3451
Slope					1		**0.8483**		0.3441		0.0027	0.0503	0.5696	0.0328	0.4164	0.2241	0.0090	0.0043	-
Surface roughness							1		0.2669		0.0031	0.0202	0.3282	0.0128	0.2343	0.1763	0.0116	0.0027	–0.0158
PRR									1		0.0708	0.0151	0.4923	0.0009	0.1222	0.0430	0.0015	0.0581	0.4012
SPI											1	0.0053	0.0101	0.0007	0.0450	0.0459	0.4962	0.6272	–0.2430
Eastness												1	0.0333	0.0163	0.1581	0.0067	0.0004	0.0017	0.0221
Hillshade													1	0.0191	0.2863	0.0966	0.0088	0.0091	0.3524
Northness														1	0.1009	0.0126	0.0032	0.0000	0.0028
Wetness															1	0.1253	0.0771	0.0279	0.0201
Altitude																1	0.0547	0.0513	–0.1574
Plan curvature																	1	0.4293	–0.0256
Profile curvature																		1	–0.0205
LST																			1

PRR represents potential relative radiation which sums daily values over the growing season. SPI represents snow potential index which indicates the snow accumulation in topography. LST represents the land surface temperature.

## Results

### Tree locations in the tree line ecotone

The overall accuracies of the tree locations classification with the IKONOS and WorldView-1 images were 96.59% and 99.44%, respectively (Table 3). The identification ability of non-tree location in the IKONOS image was better than that of tree location, whereas in the WorldView-1 image, the identification ability of the tree location was better than that of the non-tree location. This may be because trees at the upper boundary on the western side are easy to identify because of their large-diameter size. By contrast, small trees at the upper boundary of the tree line ecotone cannot be easily identified on the northern side.

**Table 3:** Classification accuracy of tree locations of IKONOS and WorldView-1 images.

		Tree class		Non-tree class		Overallaccuracy (%)
Data type	Kappastatistics	Useraccuracy (%)	Produceraccuracy (%)	Useraccuracy (%)	Produceraccuracy (%)	
IKONOS	0.9275	97.56	95.86	98.02	97.78	96.5933
Worldview-1	0.9649	99.77	98.66	99.46	98.45	99.4425

On the northern side, there is a significant upward shift trend of Mountain birch. Trees with a height ≤0.5 m at the upper boundary cannot be detected. On the western side, there are no trees showing an upward shifting trend. Compared with field observation data, trees with a height ≤0.3 m at the upper boundary cannot be detected. However, this limitation would not influence the classification results because the number of the short trees is fairly little. The upmost altitude a tree can currently reach is 2140 m asl on the northern side and 2060 m asl on western side. The upward shift of *B. ermanii* appears to promote the tree locations to high elevations on the northern side.

### Logistic regression model

The variables in the logistic regression model for the northern side of the mountains were altitude, aspect, wetness, PRR (Potential relative radiation), hillshade, SPI (Snow potential index), and slope (Table 4). Among them, altitude, SPI, and aspect contributed significantly to the regression model, which indicates that they are driving factors in determining tree locations on the northern side. The excluded variables (LST, eastness, northness, plain curvature, and profile curvature) suggested that the stand orientation and soil erosion may not influence the tree locations. It was notable that LST was excluded which may because LST has negative relationship with most of the important variables as shown in Table 2. The overall predictive accuracy of the model was 83.6% with tree predictive accuracy of 89.7% and non-tree predictive accuracy of 85.1%. For the validation area, the overall predictive accuracy was 82% with tree predictive accuracy of 79% and non-tree predictive accuracy of 85% (Table 5). The percentage of correctly predicted tree pixels was relatively low in the validation area compared with that in the training area.

**Table 4:** Logistic regression models including the model parameters of the northern and the western sides.

	Northern side					Western side			
	Coefficient	S.E.	Wald	Sig.		Coefficient	S.E.	Wald	Sig.
**Altitude**	0.002587	0.000164	**468.651**	.000	**Aspect**	0.003056	0.000495	266.497	.000
**SPI**	0.002798	0.000272	**204.687**	.000	**Wetness**	0.040304	0.000874	155.904	.000
**Aspect**	0.05056	0.000025	**169.335**	.000	**Slope**	0.011182	0.000957	79.585	.000
Slope	0.003115	0.000302	55.742	.000	Altitude	–0.002898	0.001062	42.879	.000
Hillshade	0.002969	0.000000	20.684	.001	PRR	0.001758	0.000001	20.441	.000
PRR	0.000464	0.001705	10.050	.002	LST	–0.002466	0.000258	16.967	.000
Wetness	0.000121	0.000002	6.669	.036	Plain curvature	0.050949	0.027044	6.305	.005
Constant	16.842537	0.333254	56.852	.001	Constant	3.000672	0.854591	1.393	.118
Classificationaccuracy		Observed	Predicted	Correctlypredicted	Classificationaccuracy		Observed	Predicted	Correctlypredicted
	Non-tree	11088	9782	85.1%		Non-tree	23521	20986	89.2%
	Tree	4492	4218	89.7%		Tree	4364	3704	84.9%
	Overall accuracy			83.6%		Overall accuracy			87.1%

Classification accuracy was shown with the numbers of pixels that were observed and predicted in the two classes of tree and non-tree.

The variables that have considerable effects on the models are marked in bold. Significance of all variables: *P* < 0.001.

PRR represents potential relative radiation which sums daily values over the growing season.

SPI represents snow potential index which indicates the snow accumulation in topography. LST represents land surface temperature.

**Table 5:** Classification tables showing the predictive accuracies of the complete model.

		Northern side					Western side		
Area		Observed	Predicted	Correctlypredicted	Area		Observed	Predicted	Correctlypredicted
Validation	Non-tree	44349	39201	85%	Validation	Non-tree	94081	72564	74%
	Tree	17967	15334	79%		Tree	17455	13015	72%
	Overall accuracy			82%		Overall accuracy			73%
Test	Non-tree	134893	113057	81%	Test	Non-tree	70305	55679	76%
	Tree	1863	1165	69%		Tree	1612	1326	65%
	Overall accuracy			75%		Overall accuracy			71%

Shown are the numbers of pixels that were observed and predicted in the two classes of tree and non-tree.

The variables in the logistic regression model for the western side were aspect, wetness, slope, altitude, PRR, LST, and plain curvature (Table 4). Aspect, wetness, and slope contributed significantly to the model, which indicates that they are important factors in determining tree locations on the western side. The excluded variables (eastness, profile curvature, hillshade, SPI, northness, and windthrow index) suggest that the stand orientation, soil erosion, snow cover, and wind disturbance may not influence the tree locations. Although the current tree locations are greatly affected by wind disturbance, the windthrow effect is considered only as a temporary disturbance which would not influence the tree locations. The overall predictive accuracy of the model was 87.1% with tree predictive accuracy of 84.9% and non-tree predictive accuracy of 89.2% (Table 4). In the validation area, the overall predictive accuracy was 73% with tree predictive accuracy of 72% and non-tree predictive accuracy of 74% (Table 5). Because of the wind disturbance, the tree locations scatter within the tree line ecotone, which results in low predictive accuracy in the validation area.

### Prediction of potential habitats above the tree line

**Figure 5:**
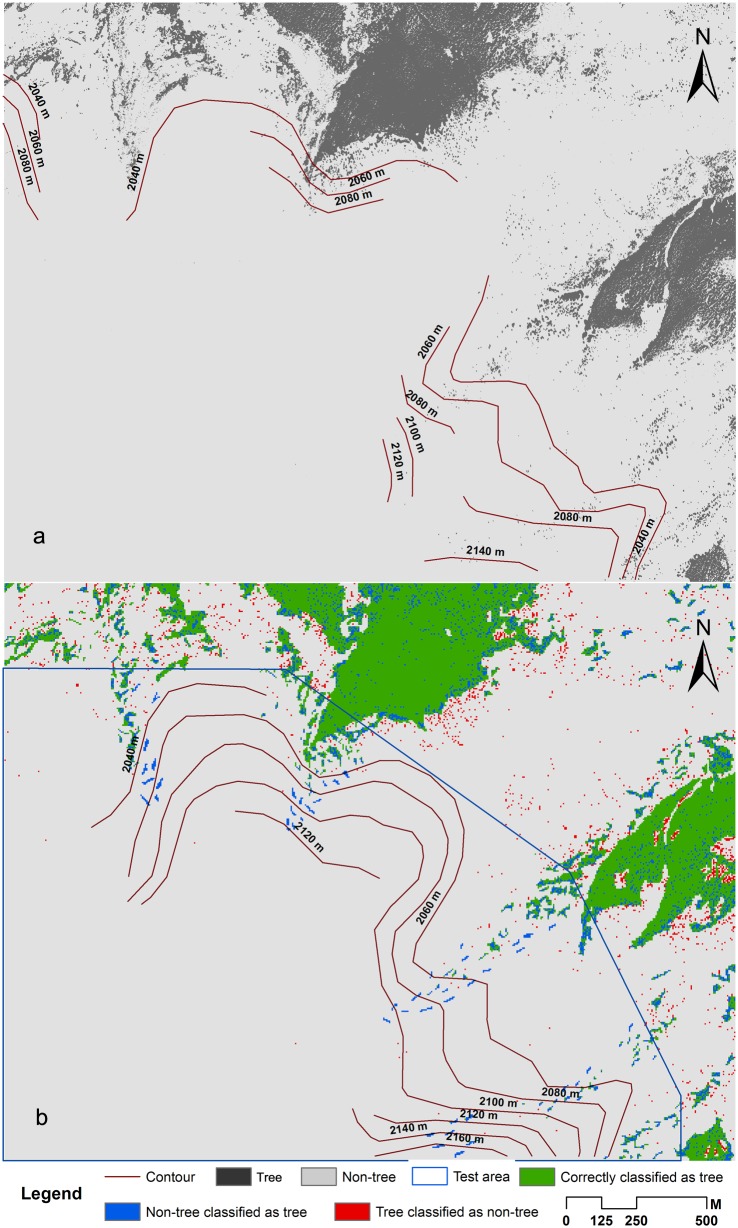
Classification and prediction maps of tree locations in the northern side. (a) Classification result of IKONOS images in the northern side. Tree classification was shown in black. Non-tree classification was shown in grey. (b) Predicted tree locations in the training area, validation area, and the test area. Green indicates correctly predicted trees. Blue indicates areas where tree is predicted where in fact tree was not present; red indicates areas where no tree is predicted where in fact tree was present.

We applied the logistic regression model to predict the potential habitats at high elevations. In the test area on the northern side, the overall predictive accuracy was 75% with tree predictive accuracy of 69% and non-tree predictive accuracy of 81% (Table 5). The model predictive accuracy was subjected to the scattered distribution of trees. The invasion of *B. ermanii* is widely accepted on the northern side. Our results indicate that there are habitats available for tree recruitments above the current tree line. Trees may potentially move to higher elevations as they have done over the past years on the northern side (Fig. 5). The upmost altitude a tree may reach is approximately 2160 m asl based on the current tree distribution features.

**Figure 6:**
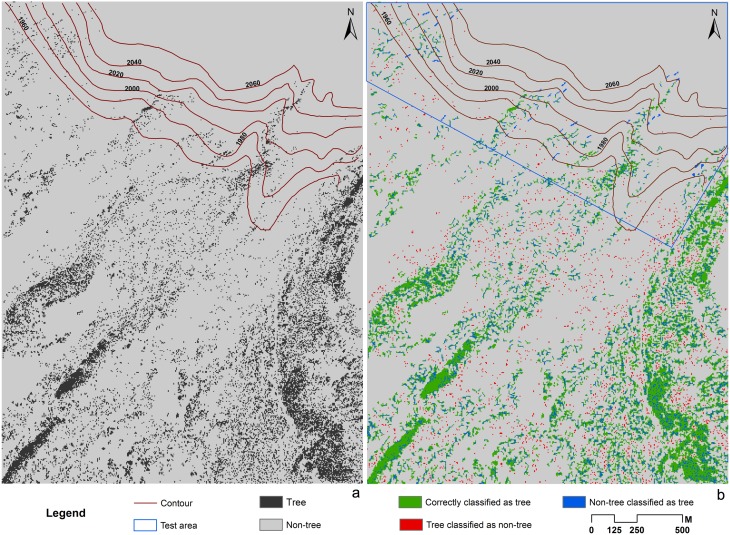
Classification and prediction maps of tree locations in the western side. (a) Classification result of WorldView-1 image in the western side. Tree classification was shown in black. Non-tree classification was shown in grey. (b) Predicted tree locations in the training area, validation area, and the test area. Green indicates correctly predicted trees. Blue indicates areas where tree is predicted where in fact tree was not present; red indicates areas where no tree is predicted where in fact tree was present.

In the test area on the western side, the overall predictive accuracy was 71% with tree predictive accuracy of 65% and non-tree predictive accuracy of 76% (Table 5). The scattered tree location greatly affected the predictive accuracy. The predicted tree location appeared to cluster in patches (Fig. 6). There are habitats available for tree recruitments above the current tree line, which indicate that trees may potentially move to higher elevations. However, the upward shifting trend of trees was not obvious. The upmost altitude a tree may reach is slightly higher than 2060 m asl, which is still lower than that of northern side.

## Discussion

### Comparison of tree locations between the northern and western sides

There are great differences in the characteristics of tree locations between the northern and western sides of the mountains. The current tree locations on the western side are subject to the windthrow effect. However, our model results show that windthrow is not the decisive factor. There is no evidence that windthrow affected tree line advance based on our field observation. Aspect, wetness, and slope had a significant influence on the tree locations. Conversely, altitude, SPI, and aspect are the dominant factors on the northern side. Environmental conditions between the northern and western sides differ mainly in the wind and precipitation regimes. Unlike the northern side, the western side is on the windward side and westerly winds prevail during the growing season [34]. Differences in the precipitation regimes may have caused differences in air and soil moisture, which contributed to the differences in tree distribution patterns. On the western side, wetness in topography affects the tree locations, which confirms our hypothesis that topographic differences are responsible for the differences in tree locations on northern and western sides of the mountains. In previous researches, scientists has attributed the variations of tree locations between the two sides to climate change and volcano activity [35], [36]. Shi and Li (2000) pointed out that the form of the tree line boundary in the Changbai Mountains was affected by the heterogeneity of micro-topography [21]. These studies confirmed the effect of topography on the local environment. However, as an important factor in the study of tree locations in tree line ecotone, temperature represented by LST data was excluded in the process of constructing the model on the northern side of the mountains, whereas it was marginally important compared to aspect, wetness, and slope on the western side of the mountains (Table 4), suggesting the effects of temperature were not significant in affecting tree location. Although temperature has been always emphasized as a major controlling factor, our study concluded that topography should be considered as an important factor in explaining the variations in tree locations in the Changbai Mountains.

### Potential tree locations

The current upmost altitude of tree location (2140 m asl) on the northern side is higher than that (2060 m asl) on the western side. We hypothesized that habitats above the current tree line are available for trees. The model prediction results confirmed our hypothesis, indicating that no matter on the northern or western side of the mountains, pioneer tree species like *B. ermanii* may potentially move to these habitats in the future. The tree lines in the Changbai Mountains have a diffused form, which is more likely to advance than those with an abrupt or krummholz form [2]. Previous studies have demonstrated that *B. ermanii* could expand its distribution range to higher elevations on the northern side, as it has expanded in the past decades [17]. Our analysis showed that *B. ermanii* could also move up on the western side, which would elevate the tree line position. However, it is noteworthy that the current position has been stable for many years on the western side of the mountains. This may be because the 1986 typhoon (∼10, 000 ha) have largely destroyed the vegetation in the tree line ecotone, which has delayed the advancement of *B. ermanii* [22]. In addition, soils in the western side of the mountains are not as developed as in the northern sides of the mountains, which could limit tree growth in the western side of the mountains [36]. Some important tree growth parameters such as biomass, age, and size, and future climate change predictions were not included in our models, which could limit our model prediction. Nevertheless, our research may help explain variations of tree locations and reveal topographic controls within the tree line ecotone in this temperate alpine region.

### Application of the high resolution image

The accuracy of the detection of tree locations depends on the spatial resolution of the available data as well as the subsequent methods [37]. Low-resolution satellite images, such as Landsat TM images, have been widely used in the detection of forest-tundra boundaries [38]. However, such macro-scale analysis often produced coarse scale results. In addition, the analysis required numerous inputs of field validation data to ensure the accuracy of the classification [39]. By contrast, high-resolution satellite images are more suitable for the detection of tree locations in the tree line ecotone [40], [41]. Unfortunately, they are difficult to acquire because of the relatively high cost compared with other commercial sensors. The IKONOS images benefit from multispectral bands and perform well in the assessment of tree locations [42]. To our knowledge, the WorldView-1 image has been rarely used in the study of tree locations because of the lack of spectral information. By contrast, the WorldView-2 (2 m spatial resolution) images with eight multispectral bands is commonly used in the extraction of tree information [43], [44]. In our study, a cloud-free WorldView-1 image could effectively identify the tree locations in the tree line ecotone. Misclassification errors in classification could be easily eliminated using few field validation data. When applying classification results to modeling analysis, particular attention should be given to eliminate the spatial autocorrelation. Otherwise, the predictive accuracy of the model will be considerably low.

In conclusion, we investigated the controlling effects of topography on the tree locations in tree line ecotone of the Changbai Mountains. Our results show that topography should not be ignored because it plays an important role in determining the tree locations. Tree locations in the tree line ecotone on the four sides (northern, southern, eastern and western) of the mountains were different. Such variations could be explained by the differences in topography. To better clarify the features of the tree locations in the Changbai Mountains, we will continue our research on the eastern and southern sides of the mountains.

## Supporting Information

Data S1 **Classification map of tree locations on the northern and western sides.**
(ZIP)
