# Supplementary figures and images for: Current and Potential Tree Locations in Tree Line Ecotone of Changbai Mountains, Northeast China: The Controlling Effects of Topography
Source: PLoS One. 2014 Aug 29;9(8):e106114. doi: 10.1371/journal.pone.0106114 (PMC4149515; doi:10.1371/journal.pone.0106114)

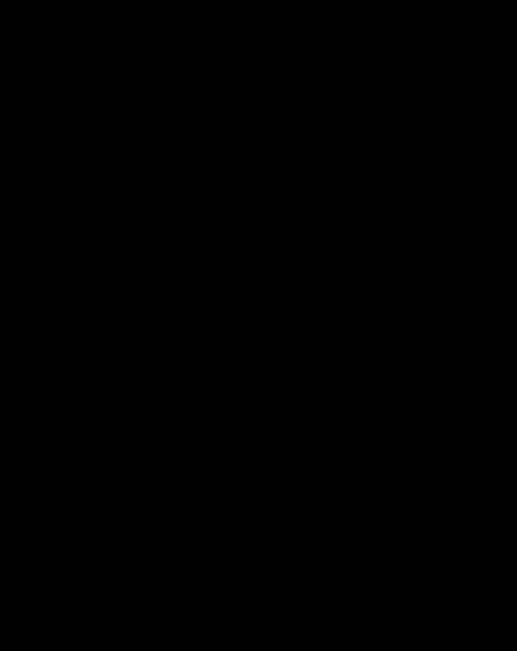

Supplement: Data S1 — Classification map of tree locations on the northern and western sides. (ZIP) [file pone.0106114.s001.zip › data/treedistributionwest.tif]

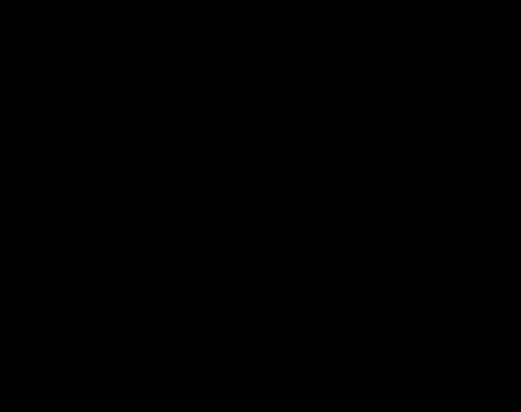

Supplement: Data S1 — Classification map of tree locations on the northern and western sides. (ZIP) [file pone.0106114.s001.zip › data/treedistributionnorth.tif]
